# Computational Modeling of Fluid–Structure–Acoustics Interaction during Voice Production

**DOI:** 10.3389/fbioe.2017.00007

**Published:** 2017-02-13

**Authors:** Weili Jiang, Xudong Zheng, Qian Xue

**Affiliations:** ^1^Mechanical Engineering Department, University of Maine, Orono, ME, USA

**Keywords:** modeling of voice production, fluid–structure–acoustic interaction, hydrodynamic/acoustics splitting method, linearized perturbed compressible equation, acoustic coupling in voice production

## Abstract

The paper presented a three-dimensional, first-principle based fluid–structure–acoustics interaction computer model of voice production, which employed a more realistic human laryngeal and vocal tract geometries. Self-sustained vibrations, important convergent–divergent vibration pattern of the vocal folds, and entrainment of the two dominant vibratory modes were captured. Voice quality-associated parameters including the frequency, open quotient, skewness quotient, and flow rate of the glottal flow waveform were found to be well within the normal physiological ranges. The analogy between the vocal tract and a quarter-wave resonator was demonstrated. The acoustic perturbed flux and pressure inside the glottis were found to be at the same order with their incompressible counterparts, suggesting strong source–filter interactions during voice production. Such high fidelity computational model will be useful for investigating a variety of pathological conditions that involve complex vibrations, such as vocal fold paralysis, vocal nodules, and vocal polyps. The model is also an important step toward a patient-specific surgical planning tool that can serve as a no-risk trial and error platform for different procedures, such as injection of biomaterials and thyroplastic medialization.

## Introduction

Voice production is a complex three-way interaction process between the glottal flow dynamics, vocal fold vibrations, and vocal tract acoustics. During voiced speech, the forced air from the lungs interacts with the adducted vocal folds to initiate self-sustained vibrations. This creates a pulsatile jet in the larynx, which is the sound source. The jet then passes through the supraglottal vocal tract, which primarily serves as an acoustic resonator to reshape the spectrum of the sound source. The acoustic pressure in the vocal tract can also propagate back to the larynx to affect glottal flow dynamics and vocal fold vibrations.

Computer models of voice production have undergone significant improvement from early lumped-mass vocal fold models (Flanagan and Landgraf, 1968; Ishizaka and Flanagan, 1972; Story and Titze, 1995; Zañartu et al., 2007) to recent continuum mechanics-based models (Alipour et al., 2000; Suh and Frankel, 2007; Zhang et al., 2007; Luo et al., 2008; Zheng et al., 2009; Šidlof and Zörner, 2013; Šidlof et al., 2015). Challenged by highly complex three-dimensional geometries and non-linear coupled nature of the dynamics, most continuum mechanics-based models have been restricted to two-dimensional, idealized/simplified geometries, or simple vocal fold kinematics. For example, many models assumed a straight rectangle tubular or cylindrical shape for the vocal tract (Zhao et al., 2002; Suh and Frankel, 2007; Luo et al., 2008; Xue et al., 2010; Mattheus and Brücker, 2011; Schwarze et al., 2011; Zheng et al., 2011a; Daily and Thomson, 2013; Jo et al., 2016). Given the wide variations found in laryngeal anatomy (Chhetri et al., 2011; Klepacek et al., 2015; Goodyer et al., 2016; Xu et al., 2016), use of such idealized geometries would impose significant constraints on the level of realism of simulations. For example, a fluid–structure interaction simulation inside a realistic laryngeal shape based on CT scans was recently reported in Xue et al. (2014). Pressure asymmetry along the longitudinal direction was observed. This asymmetry was found to be due to the oblique anterior connection between the trachea tract and larynx. It also resulted in asymmetric vibrations in the longitudinal direction, which was also observed in *in vivo* (Švec and Schutte, 1996).

The other common simplification in continuum mechanics-based models was modeling the fluid–structure interaction in the larynx and acoustic pressure propagation in the vocal tract as decoupled or separate processes (Steinecke and Herzel, 1995; Rosa et al., 2003; Duncan et al., 2006; Tao et al., 2007; Luo et al., 2008; Mattheus and Brücker, 2011; Zheng et al., 2011a,b; Xue et al., 2012, 2014; Farahani et al., 2013; Šidlof et al., 2015). These models were based on the assumption that sound generation and propagation processes are weakly coupled during normal phonation. However, recent studies have demonstrated possible strong effects of acoustic coupling on voice production (Hatzikirou et al., 2006; Zhang et al., 2006; Titze, 2008; Lulich et al., 2009; Daily and Thomson, 2013; Smith et al., 2013; Maxfield et al., 2016), pointing to the need for models to be capable of accurately predicting the acoustic coupling during voice production. A few studies used the compressible Navier–Stokes equations to model the flow and acoustic dynamics; however, they were limited to static or prescribed vocal fold motions and two-dimensional simulations (Zhao et al., 2002; Suh and Frankel, 2007; Larsson and Müller, 2009; Daily and Thomson, 2013).

The objectives of the current study were to (a) develop a first-principle-based computational model of voice production, which could faithfully model the fluid–structure–acoustics interactions with more realistic larynx and airway geometries; (b) analyze the flow waveform, vocal fold vibrations, and acoustic dynamics predicted by the model and compare them to the established data; and (c) investigate the effect of acoustic coupling on voice production.

## Computational Methodology and Simulation Setup

### Computational Methodology

The computational solver was built upon our previous immersed-boundary-finite-element method-based fluid–structure interaction solver (Mittal et al., 2008; Zheng et al., 2010; Xue et al., 2012, 2014). The glottal airflow was governed by the three-dimensional, unsteady, viscous, incompressible Navier–Stokes equations:

(1) $$\begin{array}{l} \nabla \cdot \vec{U}=0\\ \frac{\partial \vec{U}}{\partial t}+\left(\vec{U}\cdot \nabla \right)\vec{U}=-\frac{1}{\rho_{0}}\nabla P+\upsilon_{0}\nabla^{2}\vec{U} \end{array}$$

where $\vec{U}$, ρ*_0_, P*, υ_0_ are the incompressible flow velocity, density, pressure, and kinematic viscosity, respectively. The vocal fold dynamics was governed by the Navier equation with a linear stress–strain relationship:

(2) $$\rho_{tiss}\frac{\partial^{2}\vec{d}}{\partial t^{2}}=\bar{\bar{\sigma }}\cdot \overset{\leftarrow }{\nabla }+\rho_{tiss}\vec{f}$$

where ρ_tiss_ is the tissue density, $\vec{d}$ is the displacement, $\bar{\bar{\sigma }}$ is the stress tensor, and $\vec{f}$ is the body force. Details regarding the numerical algorithm of the flow and solid solvers can be found in Zheng et al. (2010).

A hydrodynamic/acoustics splitting method-based acoustics solver was integrated to realize the three-way interactions (Seo and Moon, 2006; Seo and Mittal, 2011). The acoustics field was modeled by the linearized perturbed compressible equation (LPCE):

(3) $$\begin{array}{l} \frac{\partial \rho^{\prime }}{\partial t}+\left(\vec{U}\cdot \nabla \right)\rho^{\prime }+\rho_{0}\left(\nabla \cdot {\vec{u}}^{\prime }\right)=0\\ \frac{\partial {\vec{u}}^{\prime }}{\partial t}+\nabla \left({\vec{u}}^{\prime }\cdot \vec{U}\right)+\frac{1}{\rho_{0}}\nabla p^{\prime }=0\\ \frac{\partial p^{\prime }}{\partial t}+\left(\vec{U}\cdot \nabla \right)p^{\prime }+\gamma P\left(\nabla \cdot {\vec{u}}^{\prime }\right)+\left({\vec{u}}^{\prime }\cdot \nabla \right)P=-\frac{DP}{Dt} \end{array}$$

where ρ′, ${\vec{u}}^{\prime }$, *p*′ are the acoustic perturbed flow density, velocity, and pressure, respectively, and γ is the ratio of the specific heats. The *DP*/*Dt* term represents the sound source from the flow solver. The LPCE equation is discretized with a sixth-order central compact finite difference scheme in space and integrated using a four-stage Runge–Kutta method in time. In order to resolve complex/moving geometries of biological configurations, the sharp-interface method based on the ghost-cell approach is employed for boundary treatment. Further details of this model can be found in Seo and Mittal (2011). With this splitting method, the total velocity/pressure of the flow would be the sum of the incompressible flow velocity/pressure and acoustic velocity/pressure perturbation.

The coupling process of the simulation is shown in Table 1. The fluid, structure, and acoustics solvers were explicitly coupled through a Lagrangian interface where vocal tract and vocal folds contacted. The vocal tract generally could not move except the place that contacted with the vocal folds. In each iteration, the incompressible flow was marched by one step with the existing deformed shape and velocities of the solid tissue as the boundary conditions. The acoustic solver was then marched with the updated incompressible flow field as well as the existing deformed shape and velocities of the solid tissue as the boundary conditions. The forces at the vocal fold surface were then calculated with the new incompressible flow pressure and acoustic perturbation pressure. At last, the solid solver was marched by one step with the updated surface traction. The deformation and velocities on the solid grid were then transferred to the vocal fold surface, so that the fluid/solid interface can be updated.

**Table 1:** **The coupling process of the flow, acoustics, and solid solvers**.

Step	Procedure
1	Flow solver, get the incompressible flow pressure (*P*) and velocity $\left(\vec{U}\right)$
2	Acoustics solver, get the compressible flow perturbed pressure (*p*′) and velocity $\left(\vec{u^{\prime }}\right)$
3	Update traction on vocal folds using *P*, $\;\vec{U}$, *p*′, and $\vec{u^{\prime }}$
4	Solid solver, get the tissue displacement $\left(\vec{d})\text{ and }({\vec{u}}_{tiss}\right)$
5	Deform lumen and update lumen boundary condition
6	Go to Step 1

### The Model and Simulation Setup

**Figure 1:**
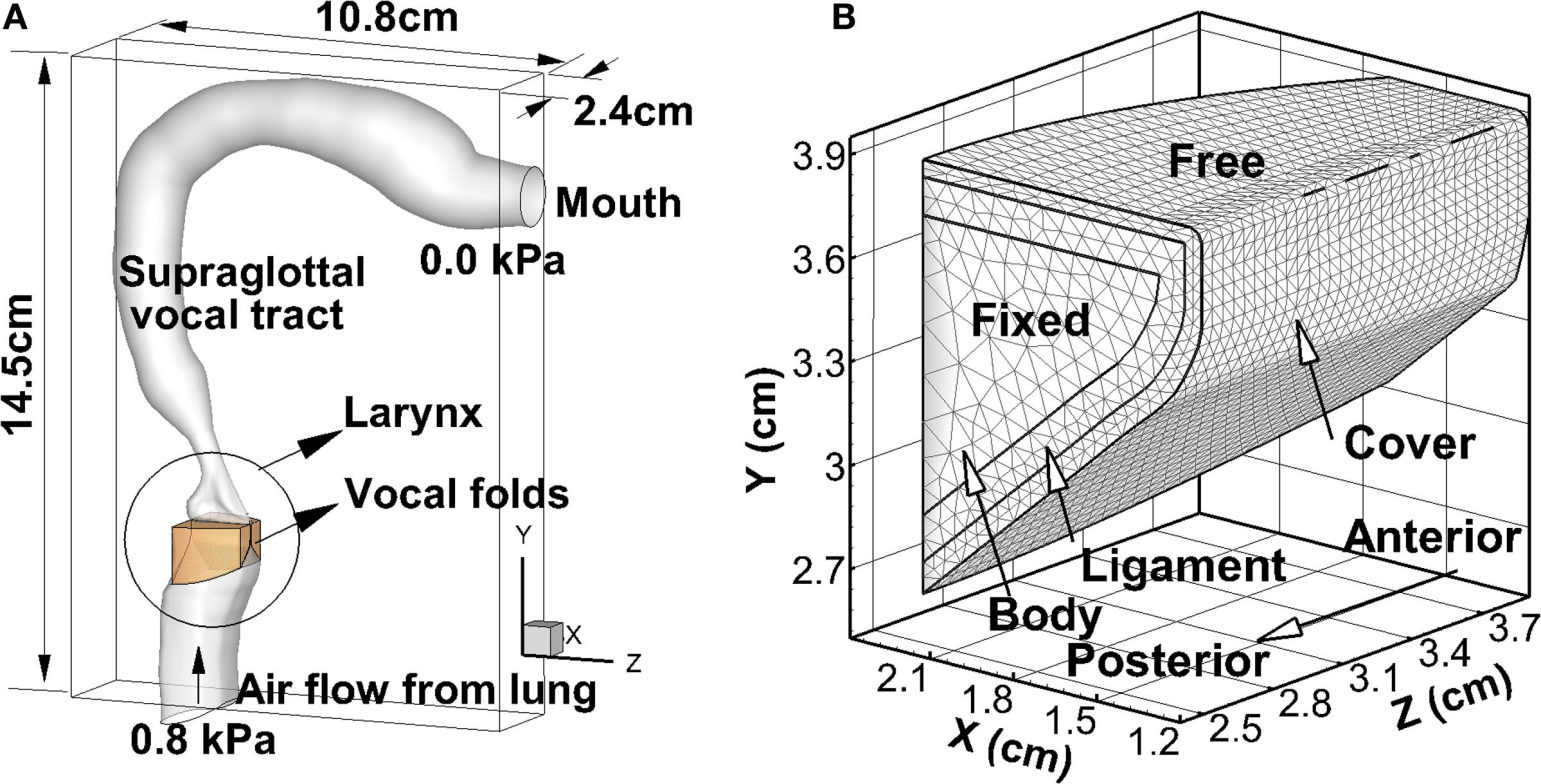
**(A)** The computational domain and geometry of the vocal folds, larynx, and vocal tract. **(B)** The inner-layer structure of the vocal fold as well as the boundary conditions applied on vocal fold walls.

The geometric model of the simulation is shown in Figure 1A. The geometry of the larynx was roughly reconstructed from a thin-slice CT scan of the larynx of a 30-year-old male subject (Zheng et al., 2009). The geometry of the vocal folds was constructed based on the mathematical model proposed by Titze and Talkin (1979), which has considered the three-dimensional shape of the vocal fold including the anterior–posterior variation. The cross-section area of the supraglottal vocal tract was taken from an *in vivo*-based neutral vowel model proposed by Story (2005), and it was superimposed onto a realistic airway center line from the *in vivo* MRI measurement (Story et al., 1996) to generate the supraglottal tract model. The length of the supraglottal tract was 17.4 cm, and the length of the subglottal tract was 3.05 cm. The vocal tract generally did not move except the place that contacted with the vocal folds.

The vocal fold was divided into three layers including the cover, ligament, and muscle (Hirano et al., 1981). Longitudinal variation in layer thickness exists (Hirano et al., 1981) but has been shown to have a negligible effect on vocal fold vibrations (Xue et al., 2011). Therefore, each layer was assumed to be longitudinally invariant in the current model. The thickness of the cover and ligament layer was 0.5 and 1.1 mm, respectively, adopted from Titze and Talkin (1979). The vocal fold tissue was modeled as viscoelastic, transversely isotropic material. The material properties (shown in Table 2) were adopted from Alipour et al. (2000) and Xue et al. (2012). Since vocal fold barely vibrates in the longitudinal direction, an in-plane motion constraint was implemented by employing relatively large values for the longitudinal Young’s moduli (Cook and Mongeau, 2007). The boundary conditions for the vocal fold are shown in Figure 1B. A zero-displacement boundary condition was applied at the anterior, posterior, and lateral surfaces, and a traction boundary condition was applied at the medial, inferior, and superior surface.

**Table 2:** **Material properties of the three inner layers of the vocal fold**.

Layer	Property						
	ρ (g/cm^3^)	*E_p_* (kPa)	ν*_p_*	*E_pz_* (kPa)	ν*_pz_*	*G_pz_* (kPa)	η (poise)
Cover	1.043	2.01	0.9	40	0.0	10	5
Ligament	1.043	3.31	0.9	66	0.0	40	7.5
Body	1.043	3.99	0.9	80	0.0	20	12.5

*ρ is the tissue density; *E_p_* and *E_pz_* are the transversal and longitudinal Young’s Modulus, respectively; ν*_p_* and ν*_pz_* are the in-plane transversal and longitudinal Poisson ratio, respectively; *G_pz_* is the longitudinal shear modulus; η is the damping ratio (Alipour et al., 2000; Xue et al., 2012)*.

A simple hard-wall contact model was incorporated to model the collision of vocal folds. Two artificial non-slip and non-penetrable collision planes were placed one grid (in this case corresponds to ±0.1 mm) off the medial plane to enforce a finite but small (0.2 mm) minimum glottal gap. Once the vocal fold reached the contact plane on its side, it would stop until forces developed in the simulation acted to push it apart. This artificial minimum glottal gap was necessary for the success of the flow solver (Mittal et al., 2008), though would result in some “leakage” flow even during what would be considered as glottal closure.

The entire geometry was immersed into a 2.4 cm × 10.8 cm × 14.5 cm rectangular computational domain (Figure 1A). For the flow model, a 0.8-kPa pressure drop was applied between the inlet and outlet. A non-penetration non-slip boundary condition was applied at the vocal tract wall. The density of air was set as 1.1455 kg/m^3^ at human body temperature. For the purpose of alleviating computational cost, the kinematic viscosity of the air was set as 6.6 × 10^−5^m^2^/s, which corresponded to approximately 1/4 of the Reynolds number of normal human phonation. Such treatment would affect the turbulence flow in the supraglottal tract that is related with high frequency effects. For the acoustics model, a hard-wall boundary condition was implemented on the vocal tract walls as $\frac{\partial \rho^{\prime }}{\partial n}=0,\;\frac{\partial p^{\prime }}{\partial n}=0,\;{\vec{u}}^{\prime }\cdot \hat{n}=0,$ where $\hat{n}$ is the outer normal vector. At the inlet, a zero Dirichlet boundary condition was applied as *p*′ = 0 and ${\vec{u}}^{\prime }=0$. At the outlet, a complete reflection boundary condition was applied as *p*′ = 0 and $\nabla {\vec{u}}^{\prime }=0$. The speed of sound was assumed to be 352 m/s.

Both the incompressible flow solver and acoustics solver employed a high resolution, non-uniform 64 × 256 × 192 Cartesian mesh, with highest grid density around the intraglottal region. The vocal fold was discretized by 28,997 tetrahedral elements. The grid was based on our experience with previous three-dimensional simulations of similar configurations (Zheng et al., 2010, 2011a,b; Xue et al., 2012, 2014; Xue and Zheng, 2016). A small time step of 1.149 × 10^−3^ ms was employed in the incompressible flow and solid solvers, while 1/20 of this value was employed in the acoustics solver to provide a good temporal resolution as well as to satisfy the CFL stability constraint. The simulation was carried out 60,000 steps on XSEDE COMET cluster, using 256 processors. The computational expense was about 15,360 CPU hours per vibration cycle.

## Results and Discussion

### Glottal Flow Waveform

**Figure 2:**
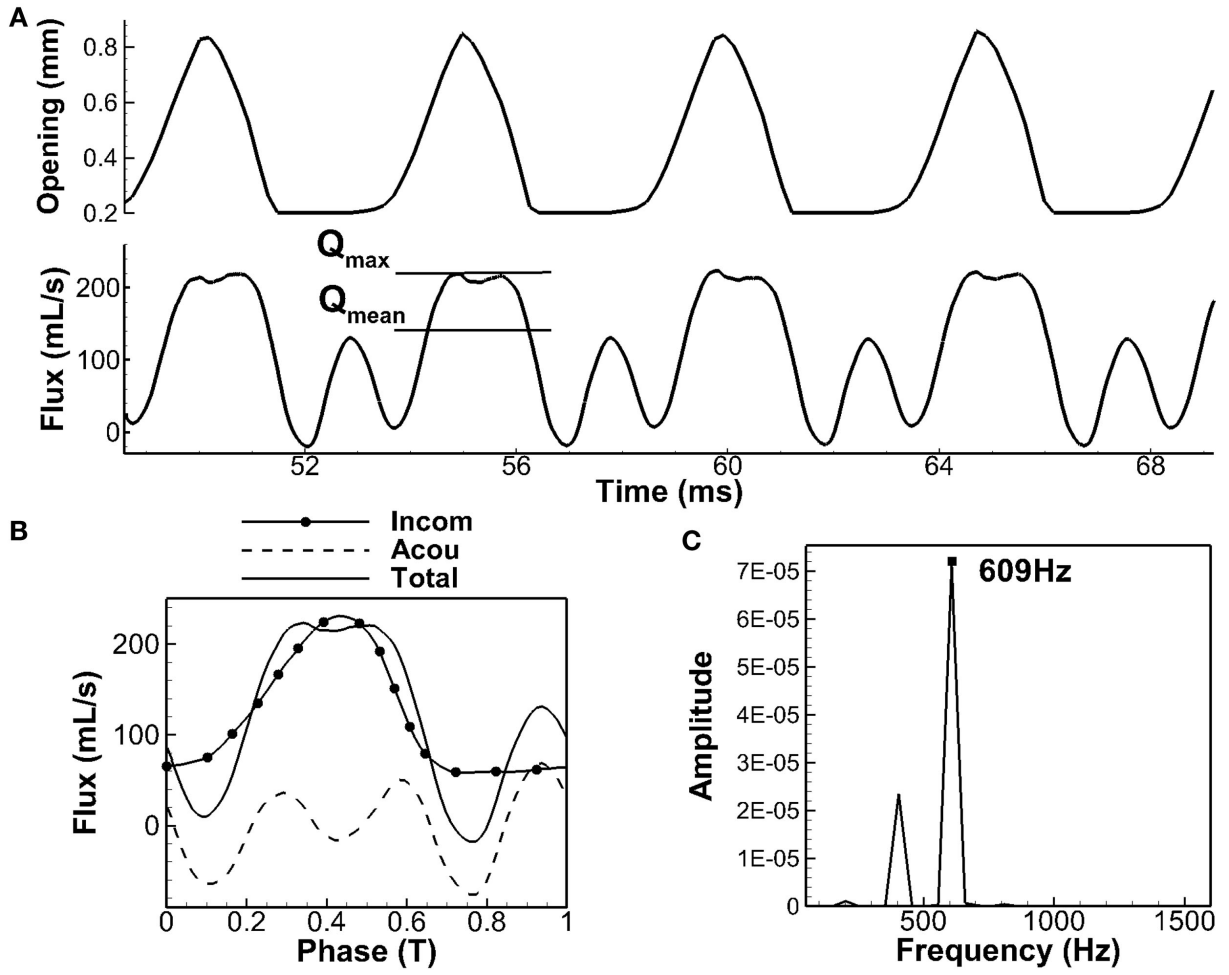
**Glottal air flow rate**. **(A)** Time history of glottal opening and air flow rate. **(B)** Phase-averaged value of total flow, decomposed into incompressible part and acoustic perturbation part. **(C)** Spectrum analysis of acoustic flow rate.

The simulation was carried out for 13 cycles, and the steady-state vibration was achieved at the 10th cycle. Figure 2A shows the time history of the glottal flow rate measured at the vocal tract outlet (mouth) as well as the opening size of the glottis during the last four cycles. The opening size of the glottis was calculated as the minimum distance between the two vocal folds at the mid-coronal plane. It can be seen that the shape of the flow rate generally followed the opening size. It increased with the increasing opening size and decreased with the decreasing opening size. It was noticed that during glottal closure (the opening size stayed at the minimum value), the flow rate did not stay at the minimum value but had a high peak. This high peak was mainly associated with the oscillation of the acoustic pressure associated with the first formant. The adopted hydrodynamic/acoustic splitting method allowed decomposing the total flow rate into the incompressible component and acoustic perturbation component, which are termed as the incompressible flow rate and acoustic flow rate, respectively, in the subsequent sections. Figure 2B shows the phase-averaged incompressible and acoustic flow rates along with the total flow rate. It can be seen that the incompressible flow rate showed a typical glottal flow waveform with slow rise and rapid fall. The total flow rate generally followed the shape of the incompressible flow rate with fluctuations due to the oscillation of the acoustic flow rate. The strong peak during the glottal closure corresponded well with the peak of the acoustic flow rate at the same phase. Strong effects of acoustic coupling on glottal flow rate was also previously observed in the model of Titze (2006a, 2008) in which the glottal flow rate was calculated using the electrical circuit analogs method (Ishizaka and Flanagan, 1972). Two to three evident ripples were generated on the flow rate waveform due to the acoustic coupling. In another work (Zañartu et al., 2007) that coupled the one-mass vocal fold model, Bernoulli flow model, and wave reflection analog to simulate the fluid–structure–acoustics interaction, the depression of the flow rate during glottal opening was observed, and this phenomenon was also shown to be due to the strong acoustic perturbation. It should be noticed that even though the glottis was closed, it was still possible to have high acoustic flow rate in the supraglottal tract due to the density change. It also needs to point out that the effect of acoustic coupling in the current model appeared stronger than in the aforementioned models. It was likely due to the total reflection boundary condition at the mouth and hard-wall boundary condition at the vocal tract wall, which had excluded the acoustic energy loss and would exaggerate the acoustic flow rate. However, such boundary conditions were considered as reasonable simplifications given small compliance of the vocal tract wall and very large area expansion at the mouth.

If the supraglottal tract is considered to be an ideal straight open-closed tube, its resonant frequencies can be analytically calculated as *F_n_* = (2*n* − 1)c/4L (*n* = 1, 2, 3, …) (Titze, 2000). For the current model with the length of 17.4 cm and the speed of sound of 352 m/s, the first three lowest formants of the tube would be 505, 1,517, and 2,528 Hz. If the area variation along the tract was considered, these values would be shifted. Story and Titze (1998) have calculated the formants of the current supraglottal tract shape with a frequency domain transmission line technique (Sondhi and Schroeter, 1987), and they found the first and second formant as 628 and 1,510 Hz, respectively. The first formant has shifted significantly due to the area variation of the tract. Figure 2C shows the frequency spectrum of the acoustic flow rate obtained from the current simulation. Its dominant frequency was 609 Hz. This value was very close to the first formant calculated by Story and Titze (1998), indicating that the oscillation of the acoustic flow rate was dominated by the first formant resonance.

Several important voice quality-related parameters were computed based on the waveforms of the flow rate and opening size of the glottis. The average values as well as the physiological range of each quantity are listed in Table 3. It was found that these values were well within the physiological range, indicating that the model had reproduced the essential biomechanics of voice production. It was noticed that the fundamental frequency (*F_0_*) approached the higher end of the physiological range, which was likely due to the large values of longitudinal Young’s moduli employed in the vocal fold model. It was of particular interest to look at the open quotient (τ_0_) and the skewness quotient (τ*_s_*). τ*_0_* is defined as the duration of the open glottis divided by the period of the cycle, calculated based on the opening size of the glottis. τ_0_ range from 0.4 to 0.7 for normal voice. A value lower than 0.4 indicates a “pressed” sound; a value above 0.7 indicates a “breathy” sound (Titze, 2000). In the current simulation, τ_0_ was 0.67, which was although within the normal range, indicated a more breathy sound. τ*_s_* is defined as the duration of flow acceleration divided by the duration of flow deceleration. This quantity was calculated based on the incompressible flow rate, as the flow acceleration and deceleration was more meaningful in the incompressible flow. τ_s_ typically ranges from 1.1 to 3.4 (Ishizaka and Flanagan, 1972; Lamar et al., 2003; Duncan et al., 2006; Luo et al., 2008). A higher value indicates that flow decreases rapidly in closing phase, which will lead to higher vocal intensity (Baken and Orlikoff, 1999; Titze, 2006b). In the current simulation, τ_s_ was 1.75, indicating moderate intensity. This was consistent with the 0.8kPa pressure drop across the larynx in the current simulation, which was typical for moderate intensity voice production.

**Table 3:** **Sound quality-related parameters**.

	Computed value	Physiological range (Xue et al., 2014)
*F_0_* (Hz)	203	60–250
*Q*_mean_ (mL/s)	143.43	110–220
*Q*_max_ (mL/s)	223.51	200–350
τ*_0_*	0.67	0.4–0.7
τ*_s_*	1.75	1.1–3.4

*F_0_ is the fundamental frequency; Q_mean_ is the mean flow rate; Q_max_ is the maximum flow rate; τ_0_ is the open quotient defined as the duration of the open glottis divided by the period of the cycle; τ_s_ is skewness quotient defined as the duration of flow acceleration divided by the duration of flow deceleration*.

### Acoustics

**Figure 3:**
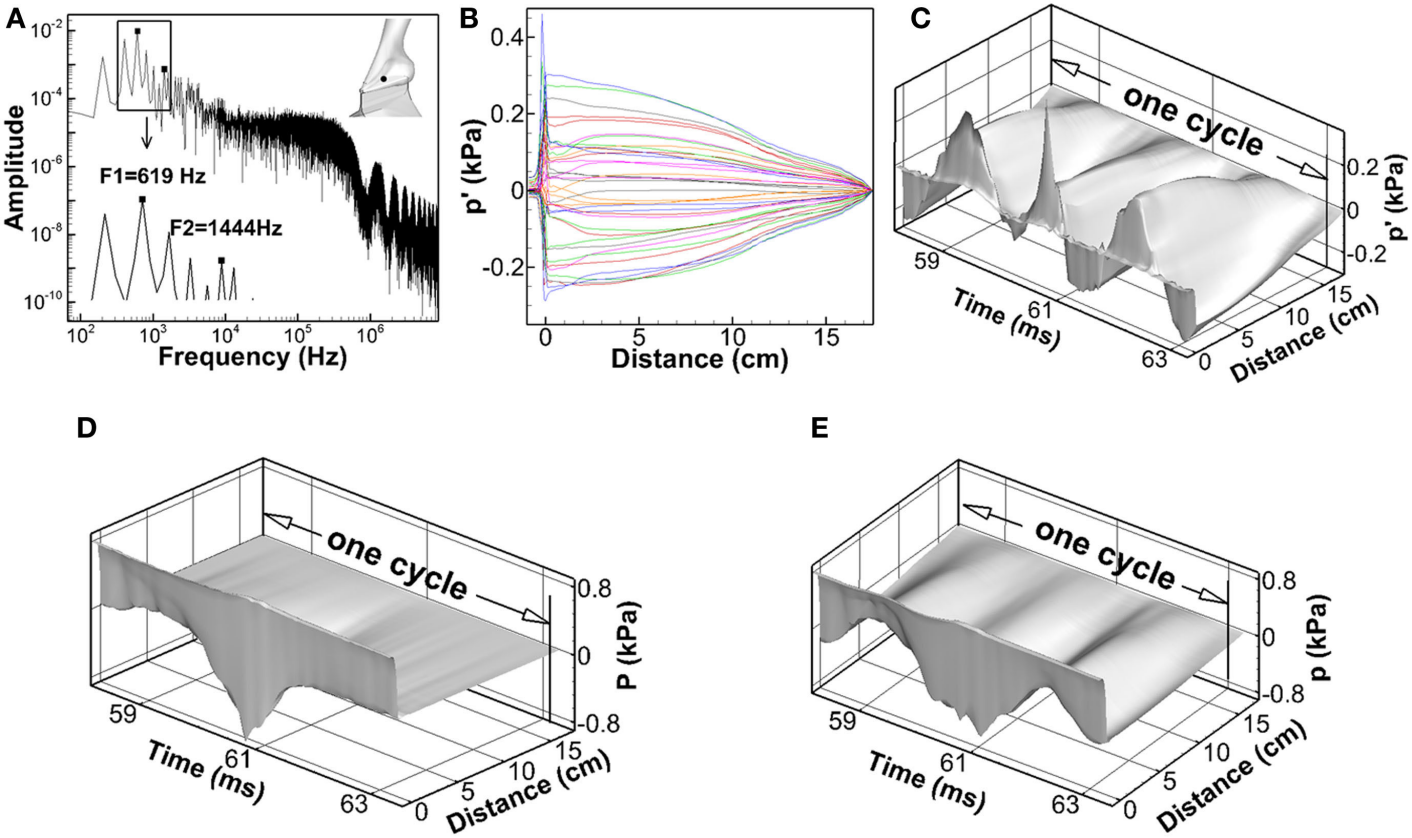
**(A)** Frequency spectrum of the acoustic perturbation pressure (*p*′) at the point of *Y* = 4.0 cm, *Z* = 3.2 cm, which was at the mid-coronal plane, and 0.1 cm above the vocal fold superior surface, indicated by the black dot in the inlaid graph. **(B)** Acoustic perturbation pressure (*p*′) along the centerline of the vocal tract at different time instants during one vibration cycle. **(C–E)** The time–spatial variation of acoustic perturbation pressure (*p*′), incompressible pressure (*P*), and total pressure (*p*) along the centerline of the vocal tract.

Figure 3A shows the frequency spectrum of the acoustic perturbation pressure (*p*′) at the point of *Y* = 4.0 cm, *Z* = 3.2 cm, which was at the mid-coronal plane, and 0.1 cm above the vocal fold superior surface, indicated by the black dot in the inlaid graph. It shows that the energy of the third harmonic, which was 619 Hz, and the seventh harmonics, which was 1,444 Hz, has been boosted due to the close distance to the first and second formants of the tract shown in the previous section. The first formant of an open–close tube generates quart-wave resonance. To verify that, Figure 3B shows the acoustic perturbation pressure (*p*′) along the centerline of the vocal tract at different time instances during one vibration cycle. Distance represents the distance from the vocal fold superior surface, with positive value corresponding to the supraglottal tract and negative value corresponding to the subglottal tract. It can be seen that a standing wave was formed within the supraglottal tract. Acoustic perturbation pressure (*p*′) oscillated in phase along the distance. The minimum vibration was at the supraglottal tract exit (distance = 17.4 cm), and maximum vibration was at the glottis exit (distance = 0). This wave shape resembled the first mode of the quarter-wave resonator. Figures 3C–E show the time–spatial variation of acoustic perturbation pressure (*p*′), incompressible pressure (*P*), and total pressure (*p*) along the centerline of vocal tract. The fluctuation of the wave amplitude of acoustic perturbation pressure (*p*′) with time was noticed. It may be because of the highly turbulent flow pattern inside the vocal tract that generated fluctuation in pressures. It was interesting to see that while the frequency of the incompressible pressure (*P*) remained same as the vocal fold vibration frequency, the frequency of the total pressure (*p*) had been largely influenced by the acoustic perturbation pressure (*p*′), especially in the supraglottal tract region. This also suggested a strong acoustic-coupling effect in the glottis.

**Figure 4:**
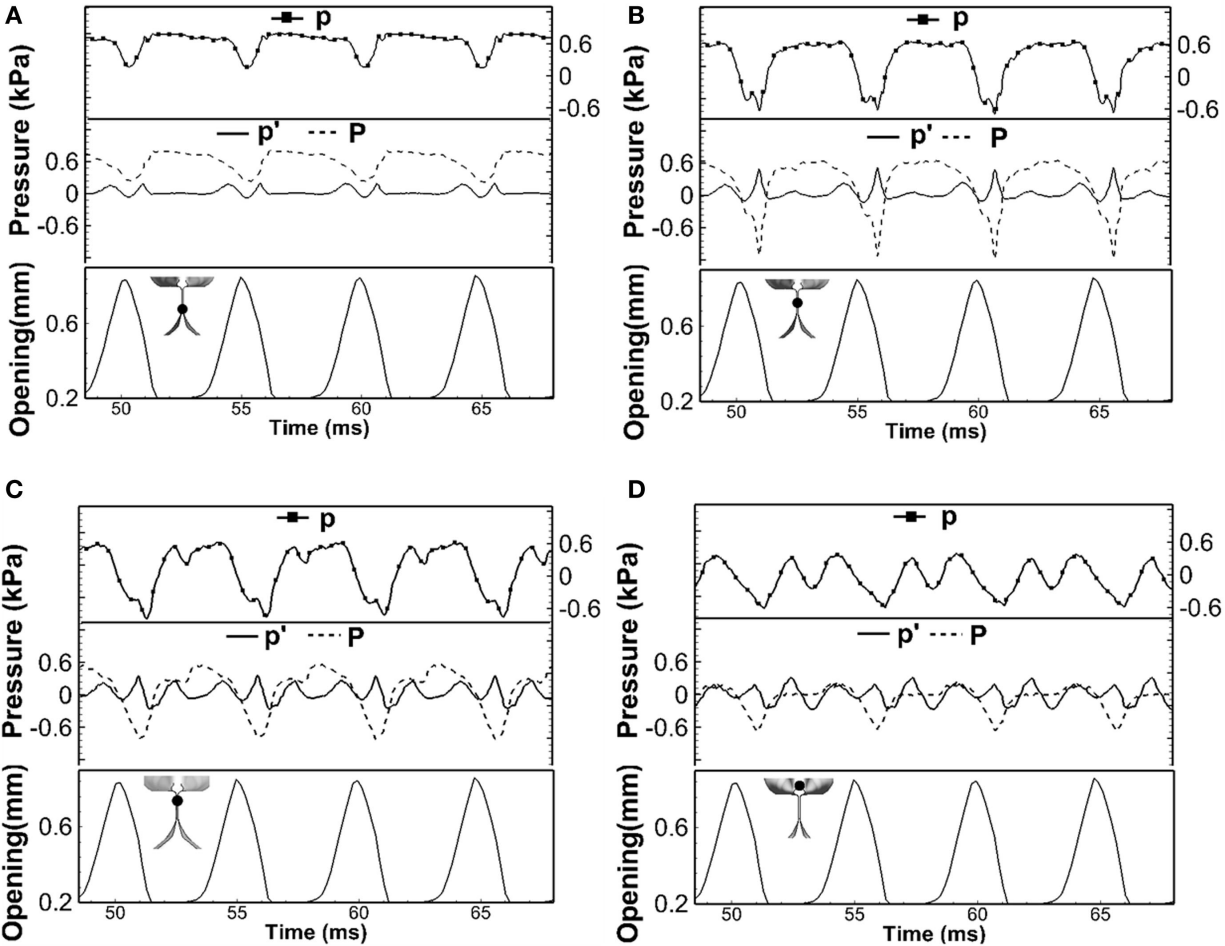
**The time variation of the total pressure (*p*), acoustic perturbation pressure (*p*′), and incompressible pressure (*P*) at four different locations: (A) *Y* = 3.6 cm, (B) *Y* = 3.7 cm, (C) *Y* = 3.8 cm, and (D) *Y* = 4.0 cm**. The locations of the points are indicated by the black dot in the inlaid graph. The variation of glottal opening is also plotted in the lower part of each subfigure.

Traditional linear source–filter theory of voice production assumes that the source–filter interaction was weak during normal phonation, and so the acoustic pressure perturbation has little influence on the vocal fold vibration (Fant, 1960; Flanagan, 1972; Stevens, 1999). This assumption is made based on the fact that the vocal fold vibration frequency is normally well below the formants of the vocal tract so that the acoustic resonance does not happen. For this case, the acoustic pressure perturbation will be much smaller than the incompressible pressure so that it had little effect on vocal fold vibrations. However, the strong effect of acoustic perturbation pressure (*p*′) on the total pressure (*p*) observed in Figures 3C and 2B suggested that this assumption may not be valid. Figure 4 shows the time variation of the total pressure (*p*), acoustic perturbation pressure (*p*′), and incompressible pressure (*P*) and at four different locations, three of which were within the glottis [(a) *Y* = 3.6 cm, (b) *Y* = 3.7 cm, and (c) *Y* = 3.8 cm] and one was just above the glottis [(d) *Y* = 4.0 cm]. The variation of glottal opening is also plotted in the lower part of each subfigure. It can be seen that, first, throughout the cycle, acoustic perturbation pressure (*p*′) and incompressible pressure (*P*) were at the same order at all positions. With the high value of acoustic perturbation pressure (*p*′), the driving force on the vocal folds as well as their vibrations will be significantly affected. Second, the total pressure (*p*) generally followed the shape of the incompressible flow pressure with fluctuations due to the oscillation of the acoustic perturbation pressure (*p*′). Third, the effect of acoustic perturbation pressure (*p*′) on the total pressure (*p*) was more significant toward the superior direction, suggesting stronger coupling effect inside the supraglottal tract. Therefore, these results suggested a strong acoustic-coupling effect during normal phonation, which may be important to be included in future modeling works. Our observation was also supported by a recent study (Maxfield et al., 2016) in which eight human subjects were recorded producing *F_0_* glide with arbitrary lengthened supraglottal vocal tract, even *F_0_* was well below the first formant of supraglottal tube, vocal fold vibration could be destabilized and resulted in *F_0_* jump.

### Vocal Fold Vibration

**Figure 5:**
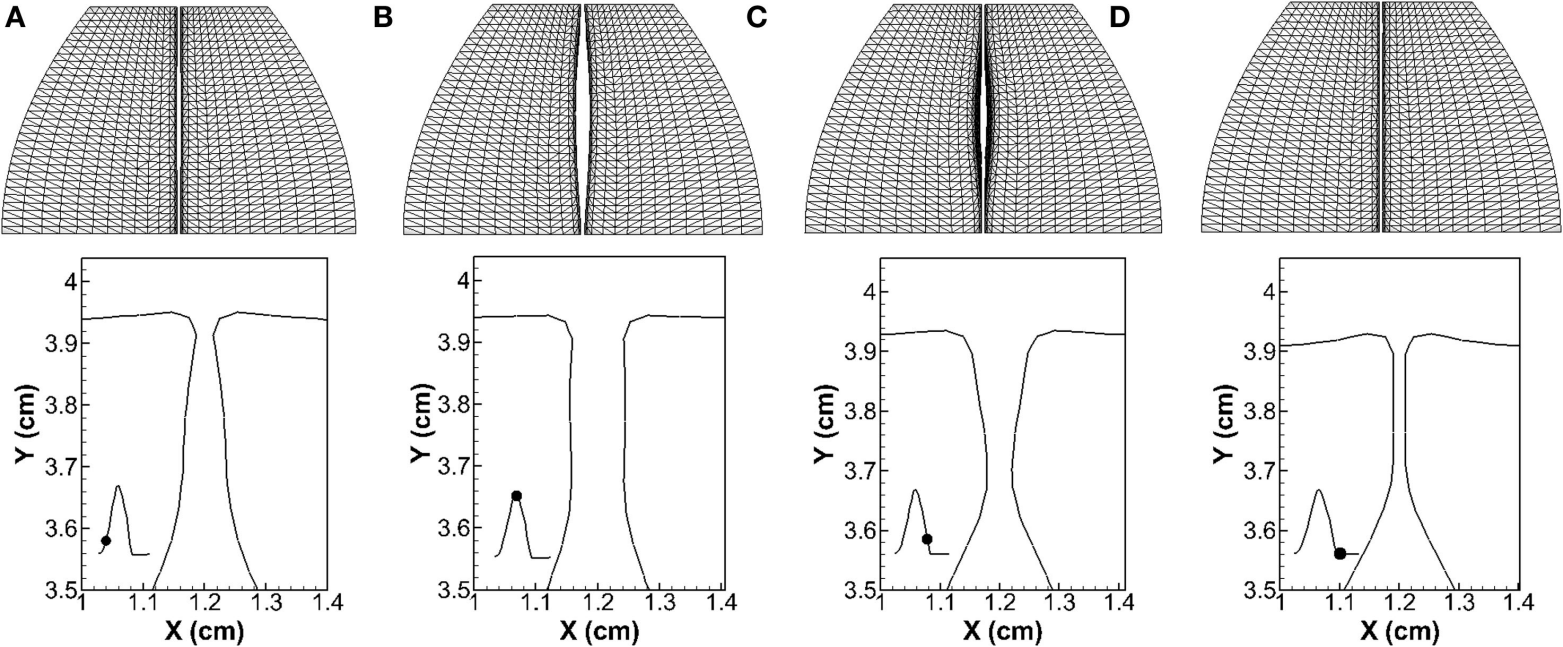
**Vocal fold vibration pattern at four time instants during one vibration cycle**. The first row shows the top view of the vocal folds, and the second row shows the profile of the vocal folds at the mid-coronal plane. The waveform of the opening size of the glottis is shown in the inlaid graph with the black dot superimposed indicating the time instant. **(A)** *T* **=** 58.7 ms, **(B)** *T* **=** 59.8 ms, **(C)** *T* **=** 60.9 ms, and **(D)** *T* **=** 61.8 ms.

Figure 5 shows the vocal fold vibration pattern at four time instants during one vibration cycle. The first row shows the top view of the vocal folds, and the second row shows the vocal folds profile at the mid-coronal plane. It can be seen that the glottis presented a convergent shape during opening (instant a) and divergent shape during closing (instant c). At instant b, the glottis reached maximum opening, and it formed a straight channel. At instant d, the glottis was fully closed with the artificial gap remained. This convergent–divergent type of motion, also called the mucosal wave propagation, is an important indicator of healthy vocalization in clinic. From the mechanical point of view, it generates a temporal pressure asymmetry inside the glottis, which ensures the net energy transfer from the airflow to vocal folds to sustain vibrations (Titze, 2000).

**Figure 6:**
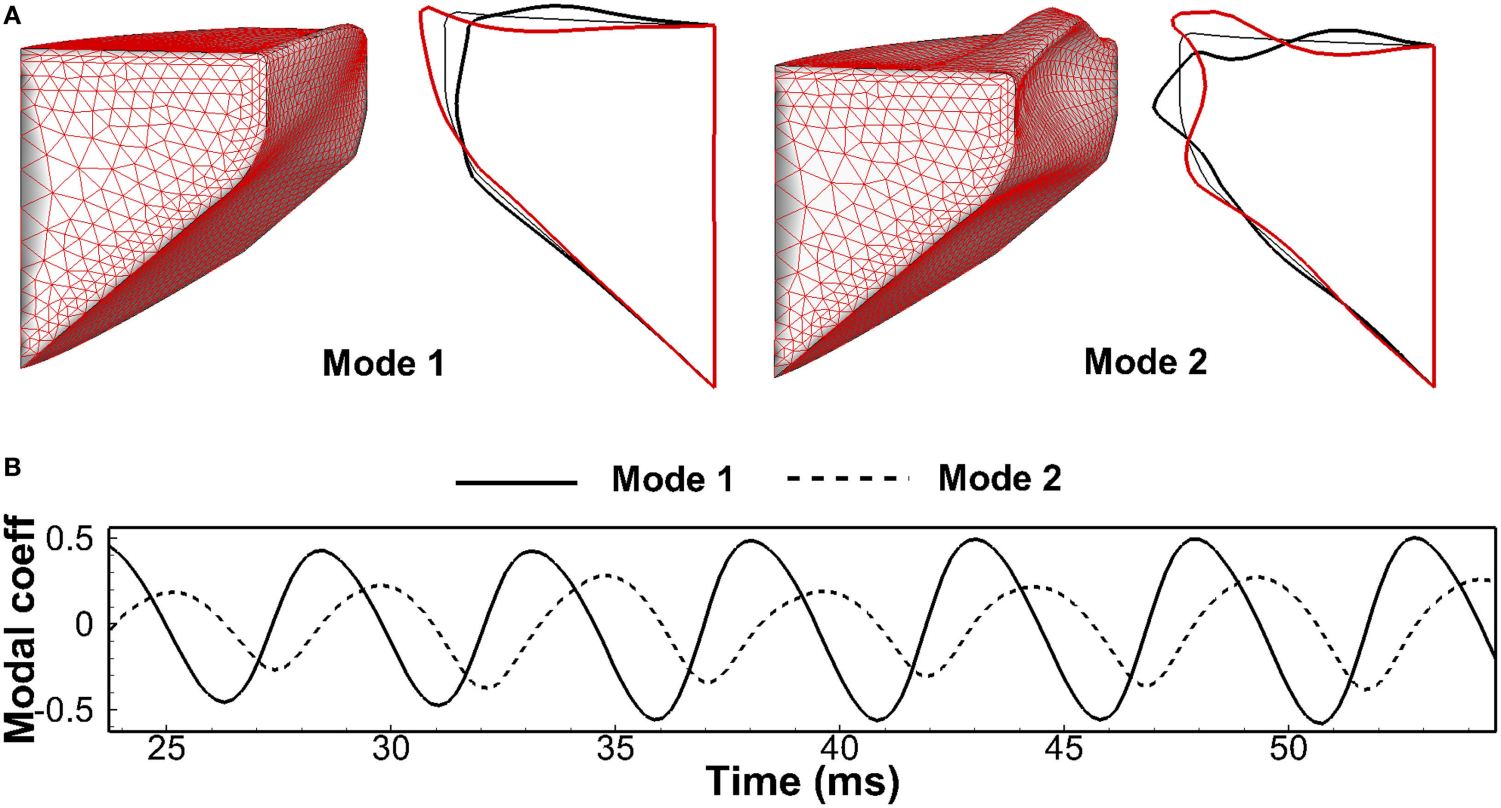
**(A)** The three-dimensional and mid-coronal profile of the most energetic two empirical eigenmodes of the vocal fold at two-extreme phases through proper orthogonal decomposition analysis. **(B)** Modal coefficients time history of the two eigenmodes.

To have a quantitative analysis, the method of proper orthogonal decomposition (POD) was utilized to extract the dominant vibratory modes (Berry et al., 1994; Zheng et al., 2011b). Figure 6A shows the two most energetic modes. Mode 1 presented a clear convergent–divergent type motion, and Mode 2 was a lateral motion. The two modes captured 92% of the total vibration energy with Mode 1 and 2 was 70 and 22%, respectively. It should be pointed out that the shape of the two dominant modes as well as the associated energy percentage were found to be very similar to Berry et al. (1994), which found that the first and second modes of vocal fold vibration in the simulation in Alipour and Titze (1985) captured 72 and 26% of the total energy, respectively. Figure 6B is the time history of the modal coefficients of these two modes. The positive (negative) coefficient of Mode 1 corresponded to a convergent (divergent) shape, and the positive (negative) coefficient of Mode 2 corresponded to glottis abduction (adduction). The two coefficients oscillated with the frequency same as *F_0_*, implying a 1:1 mode entrainment, which is an important indicator of normal phonation (Berry, 2001). Figure 6B also shows that the increasing of the coefficient of Mode 2 was always companied by the positive coefficient of Mode 1 and *vice versa*. It indicated that the convergent glottal shape formed when vocal folds opened and a divergent shape formed when it closed, which was consistent with the observations in Figure 5.

## Conclusion

The paper presented a three-dimensional, first-principle-based fluid–structure–acoustics interaction computer model of voice production, which employed more realistic human laryngeal and vocal tract geometries. Self-sustained vibrations and a reasonable glottal flow waveform were captured by the model, and important voice quality-associated parameters were found to be well within the normal physiological ranges. The important convergent–divergent vibration pattern of vocal folds was captured. POD analysis demonstrated the 1:1 entrainment of the two dominant vibratory modes. The analogy between the vocal tract and a quarter-wave resonator was demonstrated. The simulation result reflected that the acoustic perturbed flux, and pressure inside the glottis as well as the supraglottal tract were all at the same order with their incompressible counterparts, suggesting strong source–filter interactions during normal phonation.

The model demonstrated the capability of providing fully resolved and coupled flow, structure, and acoustics solutions in complex laryngeal shapes. Such a model can be very useful for developing and testing simpler models that can be transformed into clinical tools for simulation-guided diagnosis and therapy of voice diseases. It also can be useful for studying the fundamental mechanisms of voice production, especially those related to the source–tract coupling effect, turbulent sound, and different voice types. The model will greatly extend the current framework of voice modeling to a wide range of pathological conditions, which often involve complex vibration conditions.

It is also important to point out the limitations of the current model. First, the current model assumed that vocal fold tissue was linear viscoelastic material. In general, vocal fold tissue exhibits a non-linear stress–strain relationship (Min et al., 1995; Chan and Titze, 1999; Zhang et al., 2007). However, this non-linearity becomes obvious only during large deformation events such as posturing. During phonation vocal folds exhibit a nearly linear stress–strain relationship when active muscular tension is present (Titze, 2006a). Therefore, the material properties adopted in the current study can be interpreted as properties for a given posturing. Second, the current model had reduced the Reynolds number to 1/4 of the normal value to alleviate the computational cost. Such treatment would affect the turbulence flow in the vocal tract, which is related to high frequency effects. Third, an artificial gap was imposed between the two vocal folds, allowing leakage flow even during what would be considered as glottal closure. Last, the current model had employed the total reflection boundary condition at the mouth and hard-wall boundary condition at the vocal tract wall, which had excluded the acoustic energy loss and would exaggerate the acoustic flux. However, such boundary conditions were considered as reasonable simplifications given small compliance of the vocal tract wall and very large area expansion at the mouth.

## Author Contributions

WJ built the computational model, conducted the numerical simulation, and analyzed the simulation results. XZ built the computational model, modified/implemented the code for three-way flow–structure–acoustic coupling, and analyzed the simulation results. QX analyzed the simulation results.

## Conflict of Interest Statement

The authors declare that the research was conducted in the absence of any commercial or financial relationships that could be construed as a potential conflict of interest.
